# Voluntary exercise alleviates ischemic brain injury in mice by modulating mitochondrial dysfunction

**DOI:** 10.22038/ijbms.2025.80783.17488

**Published:** 2025

**Authors:** Beibei Li, Ye Zhou, Guifen Yang, Bo Li, Mingjin Zhu, Dan Lu, Senge Dai, Guoyuan Pan

**Affiliations:** 1 Tongde Hospital of Zhejiang Province, No. 234, Gucui Road, Hangzhou, Zhejiang, China; 2 The First Affiliated Hospital of Zhejiang University of Chinese Medicine, No. 54, Post and Telecommunication Road, Hangzhou, Zhejiang, China

**Keywords:** Apoptosis, Cerebral Infarction, Exercise, Mitochondria, Mitochondrial dynamics, Voluntary exercise

## Abstract

**Objective(s):**

The relationship between exercise and mitochondrial function is unclear. This study investigated the relationship between voluntary exercise and mitochondrial dynamics in ischemic stroke model mice.

**Materials and Methods:**

This experiment used 54 male C57BL/6 J mice to assess the therapeutic effect of voluntary exercise on ischemic stroke in a middle cerebral artery occlusion (MCAO) model. Body weight and the number of wheel turns were recorded to monitor the physiological condition of the mice. The degree of brain injury was evaluated via hematoxylin and eosin (H&E) staining and measurement of the cerebral infarction volume. Western blotting and immunofluorescence were used to measure dynein-1-like protein 1 (DRP1), mitochondrial fission protein 1 (FIS1), and optic atrophy type 1 (OPA1) levels to assess mitochondrial dynamics and analyze the degree of mitochondrial apoptosis by measuring cytochrome c (CYT-C), cleaved caspase-3, and caspase-3 expression.

**Results:**

Voluntary exercise positively affected the behavioral score and infarct volume. H&E staining revealed that voluntary exercise reversed MCAO-induced cortical damage. Furthermore, voluntary exercise improved mitochondrial dynamics by inhibiting DRP1 and FIS1 expression and inducing OPA1 expression. Additionally, the mitochondrial apoptosis pathway was inhibited by down-regulating the expression of CYT-C, cleaved caspase-3, and caspase-3.

**Conclusion:**

Voluntary exercise exerts a significant neuroprotective effect against MCAO-induced brain injury by regulating mitochondrial dynamics and the mitochondrial apoptotic pathway.

## Introduction

Ischemic stroke (IS), a cerebrovascular disease, accounts for approximately 85% of stroke cases (1). IS causes necrosis of brain tissue and is due to narrowing or occlusion of arteries supplying the brain. Secondary brain injury after cerebral ischemia mainly involves inflammation, oxidative stress, apoptosis, mitochondrial dysfunction, and other complex pathological processes, which eventually lead to various types of neurological dysfunction (2). Thrombolysis is currently the preferred treatment for restoring the blood supply to the brain. However, many patients do not qualify for thrombolytic therapy because they miss the treatment window or are at risk of cerebral hemorrhage. Therefore, further studies on treatment methods for IS and the mechanisms underlying this disease are urgently needed to improve IS outcomes.

Mitochondrial dysfunction is an important pathophysiological feature of IS (3). Mitochondria are the centers of energy production, play crucial roles in cells, and are closely involved in intracellular signal transduction, reactive oxygen species (ROS) production, calcium homeostasis, and other regulatory processes (4). Mitochondria constantly undergo fusion and fission to adapt to various stresses and meet the demands of cellular energy metabolism; this process is called mitochondrial dynamics (5). Fusion of the inner mitochondrial membrane is mainly regulated by optic atrophy type 1 (OPA1), which plays a key role in preserving mitochondrial ridges (5). Abnormal expression of OPA1 can lead to changes in the mitochondrial network, such as mitochondrial dysfunction and mitochondrial ridge disruption. Mitochondrial division is mediated by dynein-1-like protein 1 (DRP1). Multiple proteins located in the mitochondrial outer membrane, such as mitochondrial fission protein 1 (FIS1), are involved in the recruitment of DRP1, and overactivation of DRP1 leads to increased mitochondrial division (6).

Exercise, which is a drug-free therapeutic strategy, is an effective means of rehabilitation after brain injury and has been extensively studied. In both animal and human experiments, exercise has been shown to have a protective effect against focal cerebral ischemia. In clinical studies, long-term, individualized exercise programs were beneficial for the neurological rehabilitation of stroke patients and improved limb function, walking ability, and cognitive function (7). An MRI study revealed that exercise can activate damaged brain areas in stroke survivors and improve mobility and memory (8). In addition, exercise has multiple positive effects on rodents with stroke (9). Studies have shown that exercise can upregulate the expression of laminin and vascular endothelial growth factor, mediate cerebrovasculature formation, and promote neurogenesis (10). However, the relationship between exercise and mitochondrial function is unclear.

There has been progress in understanding the effects of voluntary exercise on mitochondrial function recovery after stroke. Previous studies by our research group revealed that aerobic treadmill training can increase the expression of translocase of the outer mitochondrial membrane 20 by activating Caveolin 1, reduce the extracellular release of mitochondrial CYT-C, protect the integrity of the outer mitochondrial membrane, and reduce mitochondrial damage (11). Mitochondrial dynamics and mitochondrial apoptosis are key factors affecting cerebral ischemic injury. However, the determination of whether voluntary exercise can help regulate mitochondrial dynamics and apoptosis and thus ameliorate biological energy dysfunction has not been studied. In this study, we constructed a cerebral ischemia model, evaluated the changes in voluntary movement, mitochondrial dynamics, and mitochondrial apoptosis, and explored the possible relationships among these three factors.

## Materials and Methods

### Animals and experimental groups

A total of 54 male C57BL/6J mice aged 8 weeks (20–25 g) were purchased from Hangzhou Medical College. The mice were housed under standard conditions (temperature: 20~25 °C; daily temperature difference <3 °C; 12/12 hour light/dark cycle; and free access to food and drink). The body weights of the mice were monitored daily. The animal experiments were approved by the Animal Experiment Ethics Committee of Zhejiang Academy of Traditional Chinese Medicine (2024, 029), and the animal experiment facility license number was SYXK (Zhejiang) 2024-0010. 

Mice were randomly divided into the following groups: 1) sham group (Sham, n = 18 mice/group), in which the mice underwent the same surgical procedure except that the internal carotid artery was not occluded and did not perform voluntary exercise; 2) MCAO group (MCAO, n = 18 mice/group), in which the mice underwent MCAO; and 3) MCAO+exercise group (MCAO+EXE, n = 18 mice/group), in which the mice were subjected to MCAO and subjected to voluntary exercise. We made every effort to minimize the number of animals and their suffering. 

### Cerebral ischemia model

The MCAO model was used in the experiment(12). Mice were anesthetized via the intraperitoneal injection of 2% tribromoethanol (T161626; Shanghai Aladdin Biochemical Technology Co., Ltd., China) at a 20 ml/kg dose. The surgery was performed as follows: 1) A longitudinal incision was made on the right side of the neck approximately 5 mm from the midline. The left common carotid artery (CCA), internal carotid artery (ICA), and external carotid artery (ECA) were carefully separated, and a small incision was made in the ECA. 2) A microfilament (L2000, Guangzhou Jialing Biotechnology Co., Ltd., China) was inserted into the ICA along the ECA until it reached the distal branch of the CCA and was ligated. Then, the muscle and skin were sutured layer by layer. 3) After one hour of obstruction, the microfilaments were removed from the CCA to allow reperfusion. 4) Finally, the mouse was placed on a heating pad and returned to its cage for regular feeding after it awoke.

Cerebral blood flow (CBF) was monitored during the modeling process via a laser speckle flow imaging system (RFLSI ZW, REWARD, China) to ensure successful modeling. A decrease in CBF of >80% was indicative of successful MCAO. Results revealed that stroke induction was successful, and model mice could be used in subsequent experiments.

### Voluntary exercise

The voluntary exercise was performed in plastic cages (28/32*18*15 cm) equipped with running wheels (diameter, 12 cm; circumference, 37.70 cm). The number of running wheel rotations was recorded via an induction pedometer. All mice were placed in cages equipped with running wheels for three days of pretraining. Formal training started on the first day after MCAO surgery and was performed daily until the seventh day when all mice were sacrificed. The exercise duration of 24 hr was recorded at 10 am every day. Mice in the other groups were placed in regular cages without running wheels.

### Behavioral test

Neurological function was evaluated with the standard of Zea Longa(13), which was a five-point scale (0–4) on the day of MCAO and on the first, third, and seventh days after MCAO (n = 10 mice/group). Scoring details are as follows: (0 points) no neurological impairments or normal limb movements; (1 point) unable to successfully extend the contralateral forepaw; (2 points) circling to the paralyzed side; (3 points) falling to the paralyzed side; and (4 points) unable to spontaneously move or walk, which resulted in a disturbance of consciousness.

### Measurement of the brain infarct volume using triphenyl tetrazolium chloride (TTC)

Mice were deeply anesthetized with tribromoethanol (T161626; Shanghai Aladdin Biochemical Technology Co., Ltd., China) and then euthanized. Fresh brain tissue was collected and cut into six coronal slices with a thickness of 2 mm(14). Slices were placed in 2% TTC solution (G1017, Servicebio, China) and incubated for 30 min in the dark before removal. A camera was used to take pictures, and the stained images were processed via Image-Pro Plus (n = 3 mice/group).

### Hematoxylin and eosin (H&E) staining

The mice were deeply anesthetized with tribromoethanol (T161626; Shanghai Aladdin Biochemical Technology Co., Ltd., China) and then euthanized. After isolation, the brain tissue was immersed in a 4% formaldehyde solution and fixed for up to 24 hr. Tissues were dehydrated in graded ethanol solutions, soaked in xylene, and embedded in paraffin. Embedded tissues were then cut at a thickness of 5 μm. Sections were subjected to H&E staining and immunofluorescence.

Paraffin sections of brain tissue were dewaxed two times each with xylene and absolute ethanol. Slices were stained blue with hematoxylin (G1005, Servicebio, China). Eosin (G1005, Servicebio, China) was then used to stain the eosinophilic structure in various shades of red (15). Samples were then sealed with neutral resin and stored. Whole brains were scanned with a pathological section scanning system (Pannoramic MIDI, Jinan Tangier Electronics Co., Ltd., China), and images were taken at ×400 magnification to observe the morphology of various nerve cells and assess the degree of nerve cell injury.

### Western blot analysis

Brain tissue containing ischemic penumbra (2 mm thick) was collected and lysed with RIPA buffer (P0013B, Beyotime, China), and the protein concentration was determined via the BCA method (P0012, Beyotime, China)(16). Proteins were separated via 10% SDS‒polyacrylamide gel electrophoresis (SDS‒PAGE) and transferred to polyvinylidene fluoride (PVDF) membranes. After transfer, membranes were incubated in 5% skim milk for two hours at room temperature and with diluted primary antibody at 4 °C overnight. The primary antibodies used were mouse anti-DRP1 (1:1000, 221099, Zenbio, China), rabbit anti-FIS1 (1:1000, R26001, Zenbio, China), rabbit anti-OPA1 (1:1000, GB115357, Servicebio, China), rabbit anti-CYT-C (1:1000, GB11080, Servicebio, China), rabbit anti-caspase-3 (1:1000, GB11532, Servicebio, China), rabbit anti-cleaved caspase-3 (1:1000, GB11532-100, Servicebio, China), and rabbit anti-β-actin (1:1000, GB15003-100, Servicebio, China). After washing with TBST, the membrane was incubated with a secondary antibody for two hours. Protein bands were photographed via an enhanced chemiluminescence (ECL) system, and the intensity of each protein band was normalized to that of the β-actin band (n = 3 mice/group).

### Immunofluorescence analysis

Paraffin sections were prepared, rehydrated, and incubated in PBST containing 5% BSA for one hour(17). Slices were incubated overnight at 4 °C with the corresponding primary antibody. Primary antibodies included mouse anti-DRP1 (1:200, 221099, Zenbio, China), rabbit anti-OPA1 (1:200, GB115357, Servicebio, China), rabbit anti-CYT-C (1:200, GB11080, Servicebio, China), and rabbit anti-caspase-3 (1:200, GB11532, Servicebio, China). Samples were incubated with corresponding secondary antibodies, including CY3-labeled goat anti-mouse IgG (1:300, GB21301, Servicebio, China) and CY3-labeled goat anti-rabbit IgG (1:300, GB21303, Servicebio, China), at room temperature for one hour and then stained with DAPI. Images were obtained via fluorescence or confocal microscopy (n = 6 mice/group).

### Statistical analyses

All data are expressed as the means±SDs, and SPSS22 software was used to analyze data. A single-factor repeated measures analysis of variance was performed to assess differences in the number of wheel turns. Weight and behavioral outcomes were analyzed by a repeated measures two-way analysis of variance, with time point and intervention as factors (time points × groups). Other data were analyzed via a one-way analysis of variance followed by Tukey’s or Dunnett’s test. *P*<0.05 indicated statistical significance.

## Results

### Number of wheel turns and body weight over time after MCAO in mice

Mice weights in each group fluctuated during and after MCAO or sham surgery (Table 1 and Figure 2 A). There was no statistically significant difference in the weights of mice at different time points within each group. In addition, there was a slight difference in body weight between the groups at each time point, but this difference was not statistically significant.

The average number of wheel turns per day in the MCAO+EXE group was recorded (day 1: 977.80±137.27; day 2: 1039.70± 153.16; day 3: 999.40±138.57; day 4: 1079.70± 138.86; day 5: 1063.40± 166.18; day 6: 1167.80± 112.07; and day 7: 1055.20± 134.66). There was no statistically significant change in the average number of daily wheel turns over time (Figure 2 B).

### Voluntary exercise enhances the functional recovery after MCAO in mice

Behavioral score data are shown in Figure 2 C. There was no significant change in the behavioral score over time in the MCAO group (day 1: 2.80±0.75; day 3: 2.40±0.66; day 7: 2.20±0.60). Over time, the neurological function scores of the MCAO+EXE group decreased significantly (day 1: 2.80±0.60; day 3: 1.90±0.70; day 7: 1.30±0.70). On day 7, the behavioral score of the MCAO+EXE group (day 7: 1.30±0.70) was significantly lower than that of the MCAO group (day 7: 2.20±0.60) (*P*=0.006 < 0.01).

### Voluntary exercise reduces the volume of cerebral infarction after MCAO in mice

TTC staining revealed that the brain tissue from the Sham group (3.74± 0.84%) appeared bright red. The brain tissue from the other two groups presented different white infarct regions of different sizes. The volume of cerebral infarction in the MCAO+EXE group (19.41±2.45%) was significantly lower than that in the MCAO group (29.08± 2.21%) (*P*=0.002< 0.01) (Figures 3 A and B). 

### Voluntary exercise alleviated brain injury after MCAO in mice

 H&E staining was used to examine tissue structure and nerve cell morphology. The brain tissue structure was complete in the Sham group; the cytoplasm was uniform, nucleoli were clear, and no inflammatory cell infiltration was observed. An irregular arrangement of neurons in the ischemic cortex, widening of the space between cells, nuclear condensation, and microglial infiltration were observed in the MCAO group. In the MCAO+EXE group compared to the MCAO group, the arrangement of cortical neurons was more regular, the space between cells was smaller, and the nucleoli were clearer (Figure 3 C).

### Voluntary exercise improved mitochondrial function after MCAO in mice

The expression of DRP1 and FIS1 was examined to investigate voluntary exercise’s effects on mitochondrial division. The protein expression of DRP1 and FIS1 in the MCAO group was significantly greater than in the Sham group (*P*=0.000 < 0.001 for both). The protein expression of DRP1 and FIS1 in the MCAO+EXE group was significantly lower than that in the MCAO group (*P*=0.034 < 0.05 and *P*=0.002 < 0.01). Immunofluorescence analysis revealed that the average optical density of DRP1 in the MCAO group was significantly greater than in the Sham group (*P*=0.002< 0.01). There was no significant difference in DRP1 optical density between the MCAO+EXE and MCAO groups (Figures 4 A-C, E, and G). 

The expression of OPA1 was measured to assess the effect of voluntary exercise on mitochondrial fusion. Western blotting and immunofluorescence analyses revealed that OPA1 in the MCAO group was significantly lower than in the Sham group (*P*=0.002 < 0.01 and *P*=0.000 < 0.001). The protein expression and average optical density of OPA1 in the MCAO+EXE group was significantly greater than that in the MCAO group (*P*=0.026 < 0.05) (Figures 4 A, D, F, and H).

### Voluntary exercise decreased mitochondrial apoptotic pathway activity after MCAO in mice

CYT-C is a key protein in the mitochondrial apoptotic pathway. The protein 

expression and mean optical density of CYT-C in the MCAO group was significantly greater than in the Sham group (*P*=0.000< 0.001 for both). The protein expression of CYT-C in the MCAO+EXE group was significantly lower than in the MCAO group (*P*=0.009 < 0.01). There was no significant difference in the CYT-C optical density between the MCAO+EXE and the MCAO groups (*P*=0.055). (Figures 5 A, B, E, and G). 

For caspase-3 and cleaved caspase-3, western blotting revealed that the protein expression of caspase-3 and cleaved caspase-3 in the MCAO group was significantly greater than that in the Sham group (*P*=0.000 < 0.001 for both). The protein expression of caspase-3 and cleaved caspase-3 in the MCAO+EXE group was significantly lower than in the MCAO group (*P*=0.021 < 0.05 and *P*=0.03 < 0.05). In addition, immunofluorescence revealed that the mean optical density of caspase-3 in the MCAO group was significantly greater than that in the Sham group (*P*=0.000< 0.001). The average optical density of caspase-3 in the MCAO+EXE group was significantly lower than in the MCAO group (*P*=0.02< 0.05). (Figures 5 A, C, D, F, and H).

## Discussion

As a cerebrovascular disease with a high incidence, IS is a leading cause of disability and death among adults in China and poses a threat to public health (18). Exercise is known to be beneficial to the human brain, as sustained exercise can accelerate the transport of nutrients and oxygen to the brain, promote brain glucose metabolism, and increase the removal of waste products from the body (19). Studies have shown that plasma injection from sedentary mice that exercise voluntarily can reduce neuroinflammatory gene expression and slow cognitive aging and neurodegeneration (20). The curative effect of rehabilitation after stroke depends not only on the intervention method but also on the motivation and initiative of patients to participate in the treatment. Therefore, in this study, voluntary wheel running was used to explore the possible positive effects of exercise on cerebral ischemia. Results showed that voluntary exercise improved functional scores, normalized histomorphology, and reduced infarct volume in MCAO model mice. It also has therapeutic significance for cerebral ischemic injury.

Mitochondria are important organelles that respond sensitively to hypoxia and play crucial roles in cell regulation. We previously reported that treadmill training can increase the expression of translocase of the outer mitochondrial membrane 20 and promote mitochondrial biogenesis after cerebral ischemia (11). A change in mitochondrial dynamics is a key biological mechanism of mitochondrial damage. The positive effects of exercise on mitochondrial dynamics have been widely reported in muscle tissue (21). However, the effects of exercise on mitochondrial dynamics in brain tissue have rarely been reported. Results of this study revealed that DRP1 and FIS1 expression increased and that OPA1 expression decreased after MCAO, which disrupted the normal mitochondrial fusion-fission balance and reflected severe mitochondrial damage. However, intervention with voluntary running significantly reversed this effect, which suggests that exercise may protect mitochondrial function by mediating mitochondrial dynamics. Amp-activated protein kinase (AMPK) is a serine/threonine kinase that controls cellular metabolism and is closely involved in regulating energy homeostasis and metabolic stress (22). Studies have reported that AMPK knockdown can inhibit the expression of DRP1 and FIS1, decrease MCAO-induced mitochondrial division, and lead to the apoptosis of nerve cells (23). Therefore, we speculate that exercise may induce mitochondrial division by regulating the AMPK/DRP1 signaling pathway, thereby improving mitochondrial dynamics and exerting a neuroprotective effect. However, further investigations are needed.

 The mitochondrial apoptotic pathway is one of the main pathways involved in ischemic brain injury and plays a key role in mitochondrial injury. CYT-C can transfer electrons in the mitochondrial respiratory chain as a carrier, establish the mitochondrial transmembrane potential, and produce ATP (24). When activated by proapoptotic proteins, CYT-C migrates from the mitochondrial membrane space into the cytoplasm and binds to Apaf-1, which activates it; the activation of Apaf-1 causes respiratory chain dysfunction and inhibits ATP generation that leads to cell apoptosis (24). Previous studies have shown that treadmill training decreases CYT-C transport from the mitochondria to the cytoplasm and inhibits the mitochondrial apoptotic pathway after cerebral ischemia (11). In addition, caspases are important components of the mitochondrial apoptotic pathway. Caspases, distributed in the cytoplasm, are a cysteine protease series with similar structures that assume an inactivated state under normal conditions. When stimulated by ischemia, CYT-C translocates from the mitochondria to the cytoplasm, where it binds to Apaf-1, recruits caspase-9 to form apoptotic bodies, activates caspase-3, and promotes apoptosis (25). In this study, CYT-C and caspase-3 levels were significantly elevated after MCAO, and voluntary exercise effectively reversed these changes. These findings suggest that exercise acts as a key modulator of mitochondrial apoptosis through a CYT-C-dependent mechanism.

**Table 1:** Body weight (g) of experimental mice groups

	Sham	MCAO	MCAO+EXE
Day 0	21.87± 1.68	22.1±1.69	22.48± 1.73
Day 1	21.04± 1.83	20.83±1.749	21.35± 1.74
Day 2	21.15±1.68	21.00±1.829	21.57±1.66
Day 3	21.43±1.77	21.25± 1.89	21.67±1.58
Day 4	22.03± 1.69	21.65± 1.69	22.01±1.48
Day 5	22.48± 1.55	21.96± 1.62	22.15± 1.65
Day 6	22.87± 1.36	22.17±1.48	22.38± 1.61
Day 7	23.36± 1.30	22.31± 1.48	22.61±1.61

Data are presented as the means±SDs.

**Figure 1:**
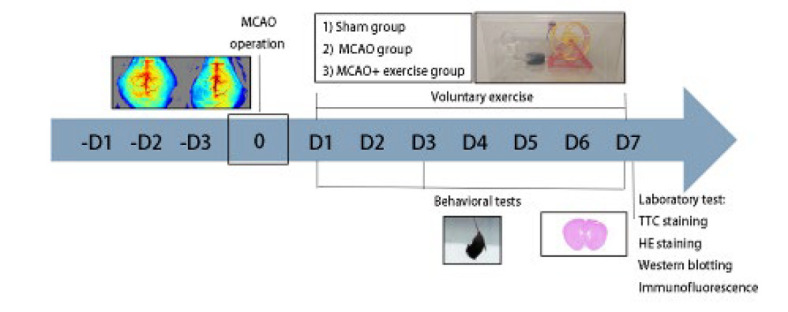
Experimental design and timeline of staining and laboratory tests on mice

**Figure 2:**
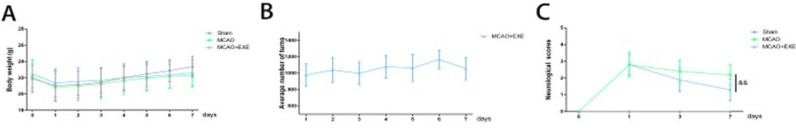
Effects of voluntary exercise on body weight of mice

(n = 10 mice/group) (A), number of wheel turns (n = 10 mice/group) (B), and the behavioral score (n = 10 mice/group) (C) are shown in the line graph. &=MCAO + EXE group vs MCAO group. MCAO: Middle cerebral artery occlusion

**Figure 3:**
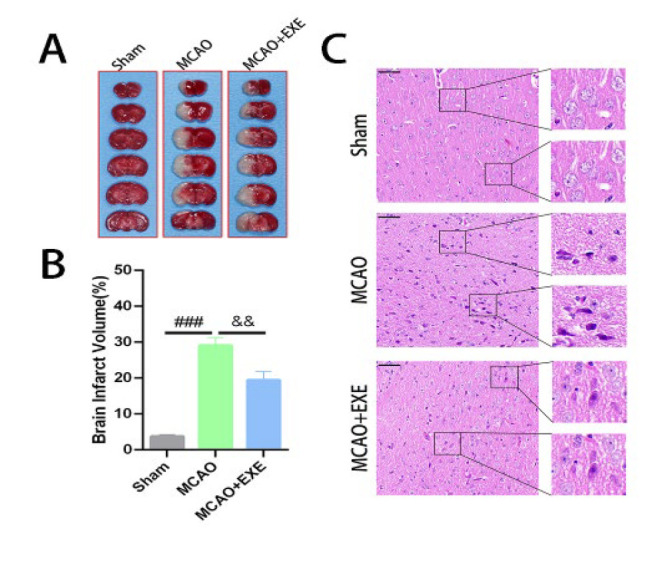
Voluntary exercise reduced the volume of cerebral infarction and alleviated brain injury in MCAO model mice

(A) TTC staining of the cerebral infarction volume, (B) A bar graph of the cerebral infarct volume (n = 3 mice/group), and (C) H&E staining revealed pathological changes (black scale=50 µm). #=MCAO group vs Sham group, &=MCAO+EXE group vs MCAO group. MCAO: Middle cerebral artery occlusion

**Figure 4:**
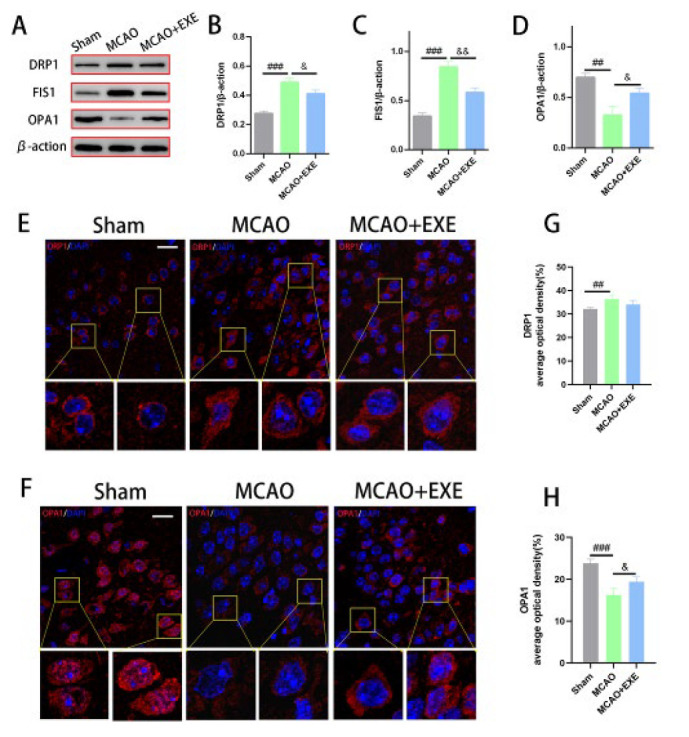
Voluntary exercise improved mitochondrial dynamics in MCAO model mice

(A) Western blot bands of mitochondrial dynamics-related proteins, (B and G) Bar graphs of DRP1 protein expression (n = 3 mice/group) and average optical density (n = 6 mice/group), (C) A bar graph of FIS1 protein expression (n = 3 mice/group), (D and H) Bar graphs of OPA1 protein expression (n = 3 mice/group) and average optical density (n = 6 mice/group), and (E and F) Immunofluorescence staining images of DRP1 and OPA1 (white scale=10 µm). #=MCAO group vs Sham group; &=MCAO + EXE group vs MCAO group. MCAO: Middle cerebral artery occlusion

**Figure 5:**
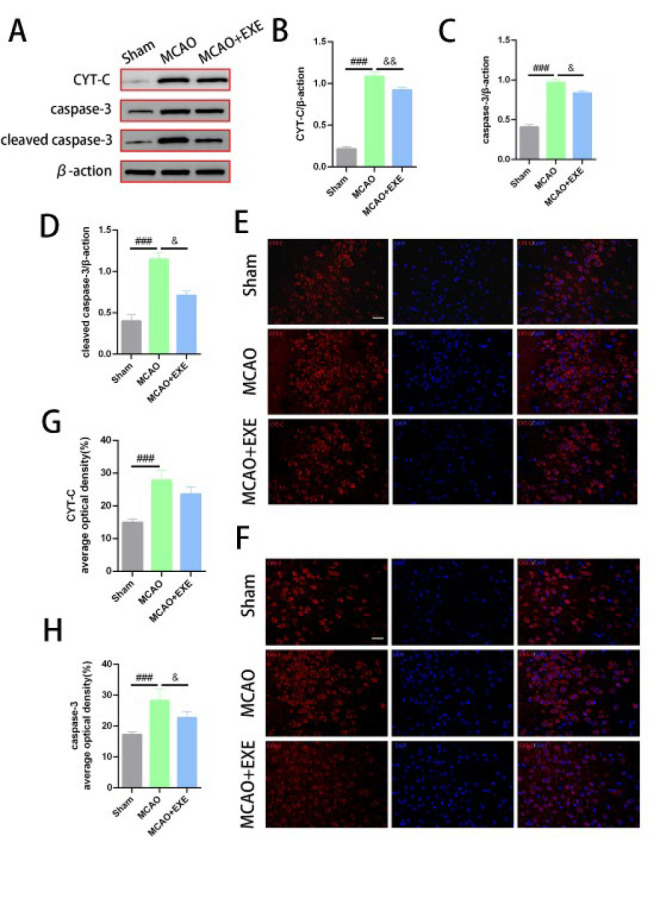
Voluntary exercise alleviated mitochondrial apoptosis in a mice MCAO model

(A) Western blot bands of mitochondrial apoptosis-related proteins, (B - D) Bar graphs of CYT-C, caspase-3, and cleaved caspase-3 protein expression (n = 3 mice/group), (E and F) Immunofluorescence staining images of the average optical density of CYT-C and caspase-3 (white scale= 20 µm), and (G and H) Immunofluorescence staining bar graphs of CYT-C and caspase-3 (n = 6 mice/group). # = MCAO group vs Sham group; & = MCAO + EXE group vs MCAO group. MCAO: Middle cerebral artery occlusion

## Conclusion

Exercise is a promising intervention for IS. It may stabilize mitochondrial dynamics by promoting mitochondrial fusion and reducing mitochondrial division. It may also inhibit the mitochondrial CYT-C/caspase-3 pathway to reduce mitochondrial apoptosis, thus exerting a neuroprotective effect after cerebral ischemia.

## Declaration

We have not used any AI tools or technologies to prepare this manuscript.
